# An Ultralight Launch-and-Recovery System for Tethered Micro Unmanned Aerial Vehicles on Small Unmanned Ground Vehicles

**DOI:** 10.3390/s26092862

**Published:** 2026-05-03

**Authors:** Yiding Liu, Zhuoqun Shen, Jingjing Xu, Sihao Chen, Bingao Zhang, Shengyong Xu

**Affiliations:** 1 Key Laboratory for the Physics and Chemistry of Nanodevices, School of Electronics, Peking University, Beijing 100871, China; 2301213327@pku.edu.cn (Y.L.); zhangbingao@pku.edu.cn (B.Z.); 2 School of Integrated Circuits, Shandong University, Jinan 250100, China; shenzq@mail.sdu.edu.cn; 3 Suzhou Xinxinlian Intelligent Technology Co., Ltd., Suzhou 215300, China; 13776337983@163.com

**Keywords:** micro-UAV, tethered UAV, UGV-UAV cooperative system, lightweight ground station, tether-assisted recovery, self-leveling platform

## Abstract

Heterogeneous unmanned ground vehicle-unmanned aerial vehicle (UGV-UAV) collaborative systems offer clear advantages for field exploration. However, when tethered unmanned aerial vehicles (TUAVs) are introduced to extend mission capability, a major compatibility gap emerges for small and highly maneuverable UGVs: existing industrial tethered ground stations are generally too heavy and bulky to be carried by such platforms. In addition, on unstructured ground, residual station tilt can significantly complicate UAV launch and recovery. To address these issues, this paper develops an ultralight vehicle-mounted tethered ground station for micro unmanned aerial vehicles (micro-UAVs) that can be integrated directly with small UGVs. Through co-design of a 2-degree-of-freedom (2-DOF) self-leveling launch platform and a passive tether-assisted recovery scheme without visual fiducials, in which a customized UAV flight-control loop is coordinated with the state transitions of the ground tether-management system, the proposed system achieves practical tether-assisted recovery. Experiments show that the complete platform weighs only 4.1 kg and that the self-leveling mechanism compensates for ground inclinations over a total range of 24 degrees. Repeated passive-landing tests further demonstrate the feasibility of the proposed recovery scheme and its tolerance to moderate bay tilt and terminal off-axis activation. System-level flight validation confirms practical tether-assisted recovery without visual fiducials. In addition, we conduct a simplified exploratory simulation of tether-based ground-anchor localization under the proposed system architecture. Overall, these results establish a lightweight and low-cost hardware design and a practically viable recovery strategy for multimodal micro air-ground robotic systems.

## 1. Introduction

Recent advances in sensor-fusion technology have made unmanned ground vehicles (UGVs) increasingly important for field exploration, geological prospecting, and related tasks [1,2,3,4]. Nonetheless, the limited sensing perspective of ground vehicles remains a major obstacle to robust autonomous navigation [5,6,7,8]. Unmanned ground vehicle-unmanned aerial vehicle (UGV-UAV) collaborative systems, also referred to as heterogeneous multi-agent cooperative systems, couple aerial and ground robots in both the physical and information domains, thereby enabling complementarity between unmanned aerial vehicles (UAVs) and UGVs in perception and task execution [9,10,11,12,13,14,15,16,17,18]. Within such systems, UAVs can rapidly acquire large-area 2.5-dimensional digital elevation models (DEMs) or three-dimensional semantic maps using onboard vision or light detection and ranging (LiDAR) sensors and transmit this global information to the ground station in real time [9,19,20,21]. The UGV can then combine these data with its own local fine-grained perception to execute reliable trajectory tracking [19,22,23]. This collaborative architecture not only shortens exploration time but also reduces collision damage and mission-failure risk for the UGV. More broadly, heterogeneous robotic systems are also increasingly discussed together with multimodal sensing and intelligent human–robot interaction paradigms, including depth-vision-based gesture interfaces and multi-sensor gesture-recognition frameworks for robot teleoperation [24,25].

Stable launch and recovery are central requirements in such collaborative systems, and miniaturization is another major concern in this study. Several factors hinder these objectives. First, many vision-based landing schemes require UAVs to carry relatively heavy vision sensors and onboard edge-computing hardware, which conflicts with micro-UAV scale constraints. Second, because small UGVs typically have short wheelbases and limited chassis size, their parking surfaces often exhibit larger residual tilt, which can significantly affect safe UAV launch and recovery if left unaddressed.

More specifically, visual approaches have already provided several solutions for landing on tilted or unstable surfaces [26,27,28]. Representative studies have extended image-based visual servoing to moving ship decks, developed lightweight onboard vision pipelines for landing support, and integrated visibility-aware perception with full-state trajectory optimization for dynamic vehicle perching. At the same time, these methods typically rely on persistent target visibility, relative pose estimation, and more complex perception-planning-control integration. Moreover, inclined landing remains dynamically challenging for conventional quadrotors because the desired terminal state is generally not an equilibrium, and additional terminal-state regulation is often required to cope with uncertainty and disturbances [29,30].

To address the launch-and-recovery constraints of collaborative systems, tethered unmanned aerial vehicles (TUAVs) have attracted growing attention as a special UAV configuration. In a tethered system, a flexible cable physically connects the UAV to the ground station. Such systems have already shown clear advantages in emergency communication, long-duration aerial lighting, traffic monitoring, and border patrol [31,32]. Compared with conventional free-flying UAVs, the presence of the tether also changes how launch and recovery should be formulated in collaborative systems. The tether introduces additional forces, geometric constraints, and controllable tension variables, so the research emphasis often shifts from open-environment target search toward coordinated control, tension management, and state estimation based on tether-related variables. Reviews and representative studies have highlighted the central role of the ground station and winch, constrained control with tether-force and tether-length limits, and localization frameworks built directly from tether geometry and tether states. These observations suggest that, in tethered systems, terminal recovery is often more naturally formulated as a constrained control problem than as a purely perception-driven landing problem [32,33,34,35].

Motivated by this distinction, the present work does not claim to replace advanced vision-based landing in general. Instead, it addresses a narrower but practically important scenario: micro-UAV deployment on small, payload-constrained UGVs, where onboard sensing and computing budgets are limited and commercially available mobile tethered stations at the relevant scale remain scarce. In our design, platform tilt is handled primarily on the ground side through a 2-DOF self-leveling mechanism and self-aligning bay geometry, while tether-assisted passive recovery is used to complete the final retrieval once the UAV has entered a coarse terminal region above the station. Mechanical platform adaptation and onboard landing algorithms should therefore be viewed as complementary rather than competing directions [36].

Accordingly, this work makes two core contributions. First, we develop an ultralight vehicle-mounted launch-and-recovery platform for tethered micro-UAVs that is compatible with the payload budget of small UGVs, combining a self-locking bay, static 2-DOF self-leveling, and compact tether management. Second, we show through ground-station/flight-controller co-design that tether tension can be converted from a disturbance into a recovery aid, making tether-assisted passive recovery practically achievable under controlled conditions. As a future-oriented extension, we also conduct a simplified exploratory simulation of tether-based ground-anchor localization.

## 2. Methods

### 2.1. System Overview

**Figure 1:**
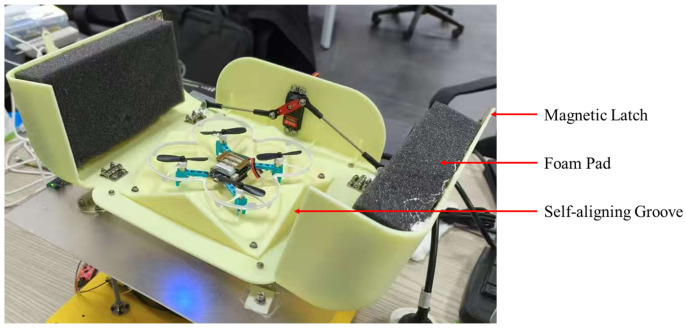
Mechanical locking and self-aligning structures. The magnetic latch and foam pads reduce impact when the bay doors are closed, while the self-aligning groove mechanically regularizes the UAV attitude after touchdown.

The system is built around a lightweight integrated mounting plate. The upper launch platform is supported at three points by one support rod and two linkages driven by magnetic-encoder worm-gear servos (ASME-SQ; sourced from Alifengsi, Tianjin, China). Vertical motion of the two servo-linkage assemblies independently adjusts pitch and roll. A laterally opening UAV bay is mounted on the launch platform, and a separate servo actuates the opening and closing linkage. Magnetic latches are installed at the interface between the two bay doors, and foam pads are added on the inner surfaces for impact absorption. Together, the 2-DOF platform, magnetic latching, and foam padding provide self-leveling capability and secure storage in non-flight conditions. In addition, to provide appropriate pose adjustment after touchdown, we added a self-aligning groove structure matched to the geometry of the UAV landing legs. As the UAV settles, the landing legs naturally slide into the recessed region of the inclined surfaces so that, together with tether pulling, the final landing pose is mechanically regularized into a limited set of stable configurations, as shown in Figure 1. After exiting the spool laterally, the tether is redirected upward by a guide pulley connected to a high-precision tension sensor (resolution: 0.001 N; Bengbu Zhongnuo Sensor Co., Ltd., Bengbu, China), passes through a guide ring at the center of the platform, enters and exits the UAV bay, and finally connects to the UAV. In addition, inertial measurement units (IMUs; MPU6050; InvenSense, Sunnyvale, CA, USA) are mounted at the corners of both the mounting plate and the launch platform to estimate platform attitude for balancing control. The interlayer naturally formed between the mounting plate and the launch platform houses the main controller board and other core control modules.

**Figure 2:**
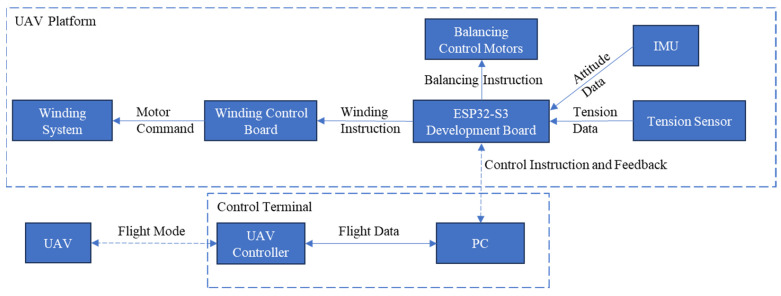
Hardware communication architecture of the overall system. The system comprises three modules, the UAV platform, the UAV, and the control terminal, with command-level communication between them.

As shown in Figure 2, the hardware and communication architecture comprises three modules: the UAV, the control terminal, and the UAV platform. The control terminal communicates with the UAV via a 2.4 GHz radio-frequency link for flight control and with the platform via Bluetooth Low Energy (BLE) for state monitoring. This study focuses on the platform side, whose control architecture is centered on an ESP32-S3 microcontroller (Espressif Systems, Shanghai, China). Serving as the platform controller, the main board integrates multisensor acquisition from the IMUs and tension sensor, parses commands from the host computer, drives the winding system through a dedicated tether-control board, and actuates the self-leveling mechanism on the basis of IMU feedback. In this way, the controller coordinates stable interaction and efficient operation across all hardware modules.

### 2.2. Tether-Management System

**Figure 3:**
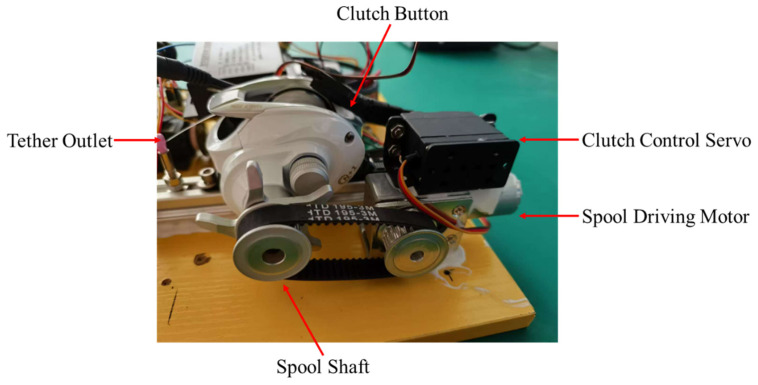
Electromechanical implementation of tether reeling and release. A clutch-enabled spool, one servo motor, and one geared motor are used to realize controlled winding and unwinding.

This section describes the tether-management mechanism and the associated tether-control board. Existing TUAV tether-control strategies include constant-tension systems [37], sensor-based closed-loop cable-management systems [35], and methods that directly couple cable tension into the flight controller for coordinated reeling [38]. Some of these approaches are too simple to generate active mechanical effects, whereas others require complex sensing architectures or substantial edge-computing resources. In our system, we design a custom clutch-enabled spool driven by a geared motor through a belt transmission, while a servo motor presses the clutch button to determine whether the spool is locked, as shown in Figure 3. We also develop a dedicated control board based on a second ESP32 chip (Espressif Systems, Shanghai, China) specifically for tether management. The board receives tether commands from the main controller through a serial interface and converts them into motor-drive instructions, thereby translating high-level commands into physical actuator states. This architecture isolates the more complex tether-management logic and exposes only a compact command interface to the main controller, resulting in a clearer control hierarchy. Table 1 summarizes the spool state-transition table defined by a finite-state machine (FSM). By defining three representative spool states and the transitions triggered by three control events, legal and illegal spool operations are explicitly formalized and embedded in the drive logic, thereby ensuring stable tether control and preventing runaway hazards. In addition to command-based control, the system provides a backup mode in which the operator can directly override the tether-management motors through the remote controller. In emergencies, the operator can forcibly stop the relevant motor to avoid more serious consequences. Compared with the methods described above, this design offers clearer separation of physical states, a more transparent hierarchical control structure, explicit exclusion of hazardous operating regions through the FSM, and a degree of passive compliance provided by the mechanical design.

### 2.3. Control-System Functions

The platform software adopts a task-based architecture to process communication, attitude stabilization, tension monitoring, and actuator control in parallel. It supports both basic and advanced system functions. Basic functions include opening and closing the UAV bay from a personal computer (PC) and manually starting or stopping the tether-management system with the remote controller. The platform also implements advanced functions that require coordinated sensing and control across multiple subsystems. For example, the self-leveling function uses the IMU mounted on the launch platform to estimate tilt angles, feeds these measurements to proportional-integral-derivative (PID) controllers, and sends the resulting commands to the balancing servos. The most important capability is the coordinated control between the tether-management system and the UAV flight controller, which enables takeoff and landing of the tethered UAV. These two advanced functions are described below.

#### 2.3.1. Self-Leveling System

**Figure 4:**
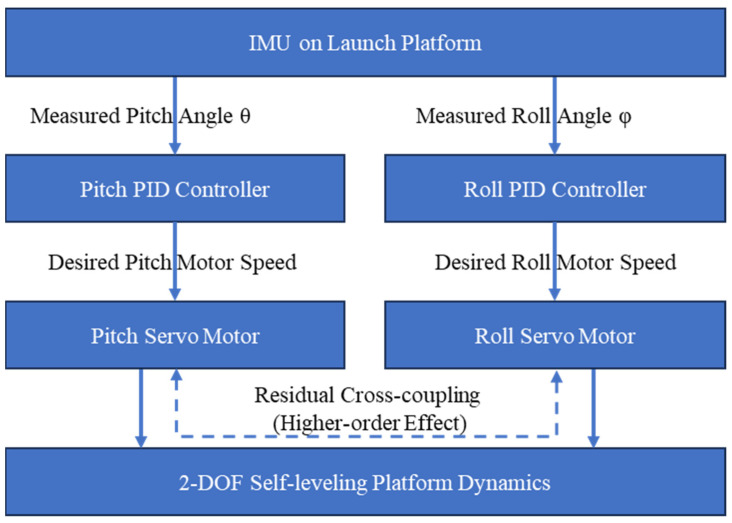
Control logic of the self-leveling system. The two servos primarily regulate platform pitch and roll, respectively. Both channels act on the same 2-DOF platform and are approximately decoupled in the small-angle operating range, while residual cross-coupling is neglected in the controller design.

In our application scenario, the vehicle is stationary during UAV takeoff, whether on flat ground or on a slope. Because structural protection against vibration and collision is already incorporated into the system, the self-leveling mechanism does not need to remain active while the vehicle is moving. Instead, the balancing mechanism is disabled and mechanically locked during motion to preserve platform stiffness. Consequently, the self-leveling system is designed primarily for static attitude adjustment rather than dynamic disturbance rejection. We use the I2Cdevlib-MPU6050 library to process data from the MPU6050 (InvenSense, Sunnyvale, CA, USA) mounted on the launch platform and to estimate pitch and roll angles. These two angles are then fed separately into their respective PID controllers, whose outputs drive the corresponding pitch and roll servos, as shown in Figure 4. Although the diagram is drawn as two parallel control channels for clarity, both channels act on the same 2-DOF self-leveling platform and share the same physical structure and sensing loop. In the small-tilt operating range (<25 degrees), each actuator predominantly regulates one attitude component, and the cross-coupling terms can be neglected to a first approximation. Therefore, the controller is implemented as two approximately decoupled SISO loops, while the residual coupling is treated as a higher-order effect [39,40]. Although the servos’ magnetic encoders can be used to set collision-avoidance thresholds, their measurement accuracy is limited (up to 3 degrees of error), which is insufficient for direct angle-command balancing control. We therefore operate the servos in velocity-control mode and feed the PID outputs into the speed command channel, thereby regulating angular velocity and achieving stable, rapidly convergent behavior. The PID parameters were tuned separately for pitch and roll, based on the decoupled two-angle control described above; the two axes followed the same procedure. The difference between the measured attitude and the target neutral-plane attitude was used as the control error. With the other two terms initially set to zero, we first increased the proportional gain until the platform responded quickly and balanced effectively when the base was tilted. The proportional gain was then increased slightly further until a small oscillation appeared after fast re-leveling, after which a small derivative gain was introduced to suppress that oscillation. Positive and negative tilts of different magnitudes were repeatedly applied to assess balancing performance, especially any visible steady-state angle error. If such an error remained, a small integral gain was added; otherwise, no integral term was used. The final tuned values depend on the exact IMU placement and the small structural and installation differences between the two linkages, so individual prototypes may require individual tuning.

#### 2.3.2. Flight-Control Redesign

To accommodate the additional variable introduced by the tether, namely tether tension, and to support the associated recovery functions, we modify the UAV flight controller. The hallmark capability of our TUAV system is passive landing. Unlike conventional precision landing, which we refer to as active landing and which relies heavily on vision or other sensors to detect the target and generate a controlled approach trajectory [41,42,43,44,45], passive landing does not require the UAV to actively search for the landing site. Instead, the UAV maintains its own stability while tether tension guides it toward the ground anchor point. We first briefly describe the UAV platform and its original flight controller. Flight-control development in this study is based on the MiniFly open-source UAV platform. The vehicle is equipped with a 9-axis IMU, a barometer, and an optical-flow module (Guangzhou Xingyi Electronic Technology Co., Ltd., Guangzhou, China), enabling velocity-command control in position-hold mode. Sensor fusion is used to estimate the current position and yaw angle, while the remote controller provides the desired trajectory and desired yaw. As a typical underactuated system, a quadrotor must change its body attitude to generate translational motion. In the original control architecture, the outer position loop converts the desired target position into desired pitch and roll angles. These references are then passed to the inner attitude loop to generate desired moments, which are combined with the desired thrust along the vertical axis to compute target motor speeds. In this way, the UAV can reach the commanded position and hover stably there.

**Figure 5:**
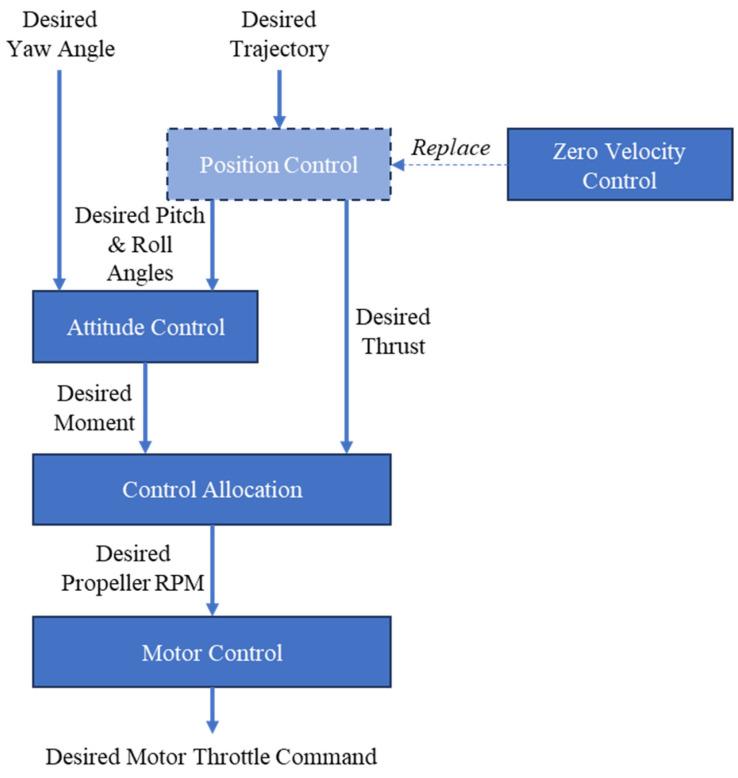
Flight-control architecture in position-hold mode. By modifying the outer position loop, we implement a passive-landing mode that cooperates with tether tension.

This control architecture becomes problematic during passive landing. Because the position loop continuously anchors the UAV to a fixed three-dimensional coordinate, any tether-induced displacement from that coordinate during hover is strongly opposed by the controller, which can destabilize the vehicle. A new mode is therefore required to decouple stabilization from absolute position holding so that the UAV can move gradually under tether action while maintaining attitude stability and avoiding loss of control. To this end, we replace the original position loop with a zero-velocity feedback loop. Specifically, the translational velocity of the UAV is estimated and compared with zero, and the resulting error is fed to a PID controller instead of using position error as the control input. Under this scheme, the UAV actively suppresses its own translational speed while retaining stable flight. As landing proceeds, the altitude decreases gradually. Once the altitude falls below a preset threshold (in our implementation, below 15 cm for more than 0.3 s), the UAV executes the landing routine and progressively shuts down the propellers. During parameter tuning, we first tried directly reusing the PID gains from the original position loop in the zero-velocity loop, but the controller became clearly unstable and quickly lost control. We then slightly increased the proportional gains in the two horizontal axes and substantially increased all three gains in the vertical axis, eventually obtaining a zero-velocity-loop mode that could both hover stably and be passively displaced by tether reeling. During this tuning process, we also observed how larger vertical-axis PID gains changed the landing trajectory, which motivated the theoretical analysis presented in Section 3.3.1. Figure 5 illustrates the modified flight-control architecture.

## 3. Results

To evaluate the proposed system, we first provide an application-oriented scenario illustration and an illustrative market survey to clarify the intended operating context. We then assess the self-leveling and passive-landing functions, analyze the mechanism underlying passive landing and its benefits, and conduct system-level flight validation. Finally, as an exploratory study, we conduct a simplified simulation to examine whether tether-direction information could potentially support ground-anchor localization.

### 3.1. Scenario Illustration and Illustrative Market Survey

**Figure 6:**
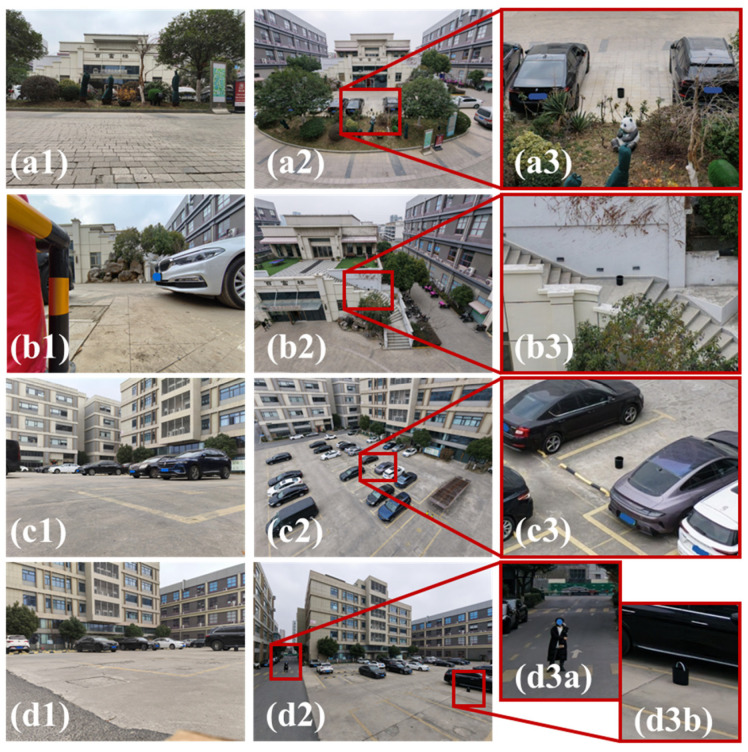
Application scenario illustration for a small UGV-UAV collaborative system. In each group, (**a1**–**d1**) shows the camera view from the ground vehicle, (**a2**–**d2**) shows the UAV camera view after deployment, and (**a3**–**d3**) shows a magnified view of the corresponding aerial image. In (**d3**), (**d3a**) and (**d3b**) show enlarged views of the experiment personnel and the target object located at two ends of the scene, respectively, indicating that the UAV viewpoint can observe both simultaneously. The non-English text visible on the building facade is a real-world building/complex name in the photographed environment and is not used as an experimental label. The figure is intended to illustrate the use scenario of aerial-ground collaboration rather than to validate the proposed flight-control or recovery method.

Before evaluating the core subsystems, we first clarify the intended application context of the proposed platform. To illustrate the role of aerial deployment in a small UGV-UAV collaborative system, we recorded ground and aerial views in several representative outdoor scenes after mounting the platform on a small UGV. For this scenario-illustration experiment, we used a commercial DJI Neo 2 micro-UAV (DJI, Shenzhen, China) equipped with a 4K camera, since it is dimensionally compatible with the platform and can be deployed without additional structural modification. An auxiliary camera on the UGV was used to record the ground perspective. As shown in Figure 6, the aerial viewpoint provides additional scene context in situations involving low obstacles, local height differences, or more complex path layouts. This experiment is intended only to illustrate the collaborative use scenario and the motivation for carrying a micro-UAV on a small UGV; it is not used to validate the flight-control redesign or passive-landing method. All subsequent control and recovery experiments were performed on the MiniFly-based tethered prototype.

We further conducted an illustrative market survey of representative commercial UAV ground stations, with particular attention to tethered systems, as summarized in Table 2. The purpose of this survey is to clarify the current engineering landscape rather than to provide a strict like-for-like benchmark against our prototype. During the survey, we did not identify commercially available mobile ground stations specifically designed for micro-UAV deployment on small UGVs. Consequently, the representative products listed in Table 2 mainly correspond to larger UAV classes. We therefore include them not as direct performance baselines, but as evidence of a current product gap between existing commercial systems and the small-UGV/micro-UAV scenario considered here. This gap also highlights the exploratory value of pursuing a mobile ground station at the micro-UAV scale. Against this background, the proposed prototype occupies a different design point, with an integrated size of 300 × 300 × 260 mm and a total mass of 4.1 kg, and is intended for direct mounting on small UGVs with limited payload budgets. In this sense, Table 2 should be read as a gap-oriented market survey rather than a direct performance comparison across different UAV classes.

### 3.2. Performance Evaluation of the Self-Leveling System

The self-leveling system is critical for ensuring that a ground station parked on unstructured terrain can reliably support UAV takeoff and landing, and its performance therefore requires quantitative evaluation. In practical operation, the self-leveling system is activated after the UGV stops and compensates for the platform attitude caused by ground inclination, thereby providing a safe launch and recovery surface. After mission completion and successful UAV recovery, the self-leveling mechanism returns to its neutral position and is mechanically locked to prevent vibration damage during subsequent UGV motion.

**Figure 7:**
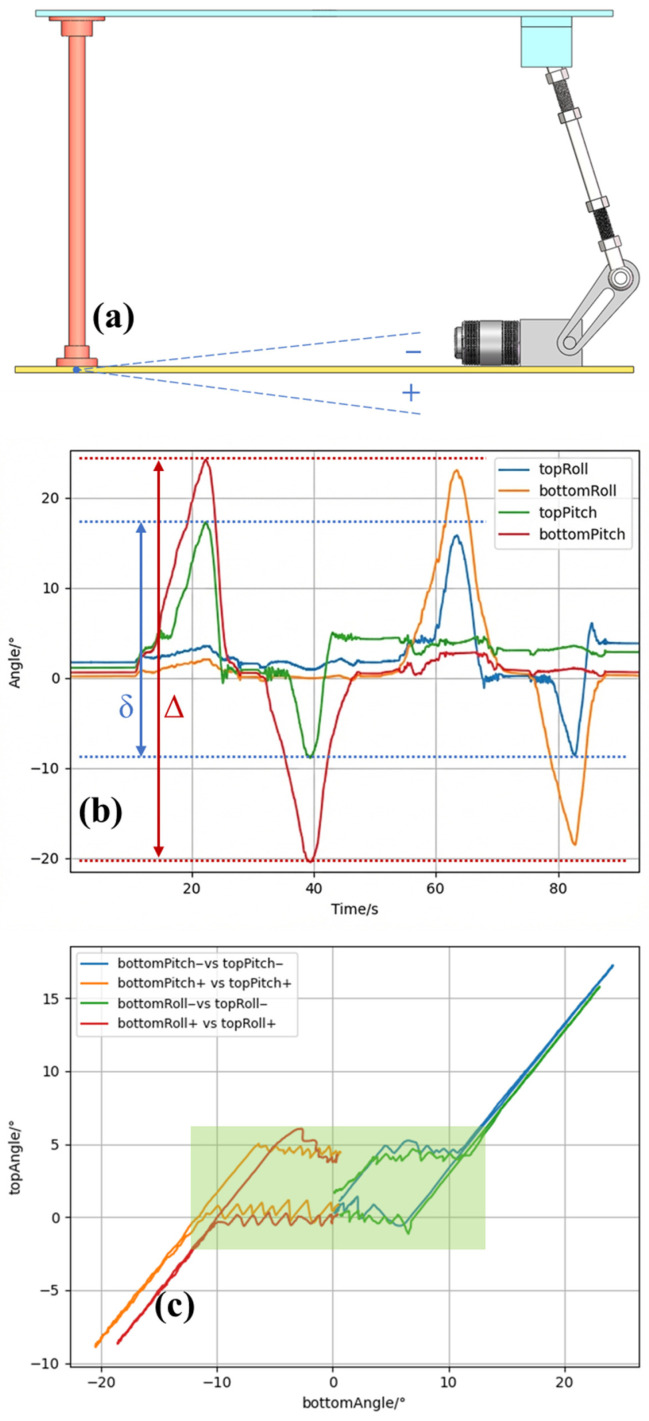
Static tilt experiment. (**a**) Definition of the positive and negative tilt directions. (**b**) Time histories of roll and pitch measured by the two IMUs during the experiment. (**c**) Phase plot relating the corresponding IMU measurements. The green region in (**c**) denotes the full-compensation region under the 7.5° allowable tilt-error band defined in the text.

We designed a static tilt experiment to evaluate system performance under different inclinations. The platform was slowly tilted manually beyond 20 degrees in both the pitch and roll directions and then returned, and the same procedure was repeated in the opposite direction. Throughout the experiment, pitch and roll measurements from the IMUs mounted on the mounting plate and the launch platform were recorded. Figure 7a defines the positive and negative directions used in the test: when the distance between the upper-plate linkage connection point and the bottom plate is smaller than that at the neutral plane, the corresponding motion is defined as negative; otherwise, it is defined as positive. Figure 7b presents the time histories of the pitch and roll angles measured by the two IMUs, where the horizontal axis denotes time and the vertical axis shows topRoll, bottomRoll, topPitch, and bottomPitch. Figure 7c gives the corresponding phase plot, with bottom IMU pitch and roll on the horizontal axis and top IMU pitch and roll on the vertical axis; the positive and negative labels in the legend follow the sign convention defined in Figure 7a. Because of slight installation offsets, direct inspection showed that both IMUs corresponded to a truly horizontal platform when their pitch and roll readings were approximately 2.5 degrees; this state was therefore defined as the neutral plane. To improve practical stability near level ground, we introduced an allowable tilt margin of ±3° around the neutral plane, within which the motors stop moving. This setting proved useful in practice because it preserves sufficient leveling accuracy while enhancing static stability. As shown in Figure 7b, when the platform is tilted slowly and the inclination is small, the top-IMU readings follow those of the bottom IMU because the tilt remains within the allowable margin and the actuators stay still. Once the inclination moves beyond the ±3° margin and continues to increase, the top-IMU readings are regulated near the edge of the allowable region in a sawtooth-like manner, indicating that the self-leveling mechanism is repeatedly adjusting the platform attitude to drive it back into the admissible range. When the ground tilt increases further, the linkage reaches its compensation limit; beyond this point, the top-IMU readings rise together with the bottom-IMU readings while maintaining an approximately constant amount of tilt reduction. In this experiment, the total variation in the bottom IMU readings across the experiment is indicated by the spacing of the red dashed lines, whereas the corresponding variation in the top IMU readings is indicated by the spacing of the blue dashed lines. Because the bottom IMU is mounted on the base plate and directly reflects ground inclination, these results indicate that when the ground tilt amplitude is below 22.29 degrees, the self-leveling system reduces the tilt amplitude by at least 41.28%. Figure 7c further clarifies the balancing mechanism: within a certain range, the platform constrains the launch surface attitude to remain within a bounded region, and beyond that range it functions as a roughly constant compensator that reduces the effect of additional tilt. Because an allowable margin is intentionally introduced, the relation between the bottom- and top-IMU angles is not one-to-one, and hysteresis-like loops appear depending on the direction from which the platform tilt re-enters the allowable range. If an allowable tilt error band of 7.50 degrees (obtained by adding 1.5 degrees to the 6-degree control margin to accommodate small overshoot) is defined as the horizontal state and represented by the height of the green region, the system can fully regulate a total tilt range of approximately 24 degrees, represented by the width of the green region. Taking the above neutral plane as the true horizontal position, the system can therefore fully compensate for tilts within ±12 degrees. Even beyond this range, the mechanism still provides roughly 12 degrees of tilt reduction.

### 3.3. Passive Landing and System-Level Validation

This subsection examines the principle and performance of passive landing and verifies the overall operating workflow. As noted above, passive landing brings the UAV to the ground tether anchor by reeling in the tether. Unlike conventional active landing, the UAV does not need prior knowledge of the target’s relative position, nor does it need to actively generate an approach trajectory through motion planning or visual servoing. Because the method does not rely on vision, it substantially reduces onboard computational demand and eases the burden on edge computing, which is advantageous for further UAV miniaturization.

In our implementation, passive landing requires coordinated operation of the UAV and ground platform. After the PC issues a passive-landing command, the UAV switches to passive-landing mode while the tether-management system simultaneously enters the winding state. When the UAV detects that its distance to the platform below has fallen beneath a preset threshold, it automatically initiates gradual propeller shutdown and landing. When the tension sensor detects either a sudden rise in tension or a value above the safety threshold, the system interprets this as evidence that the UAV has already come to rest inside the bay. The tether-management system then immediately stops winding and actuates the clutch to release the internal tether tension. At that point, the passive-landing process is complete.

#### 3.3.1. Trajectory Analysis

**Figure 8:**
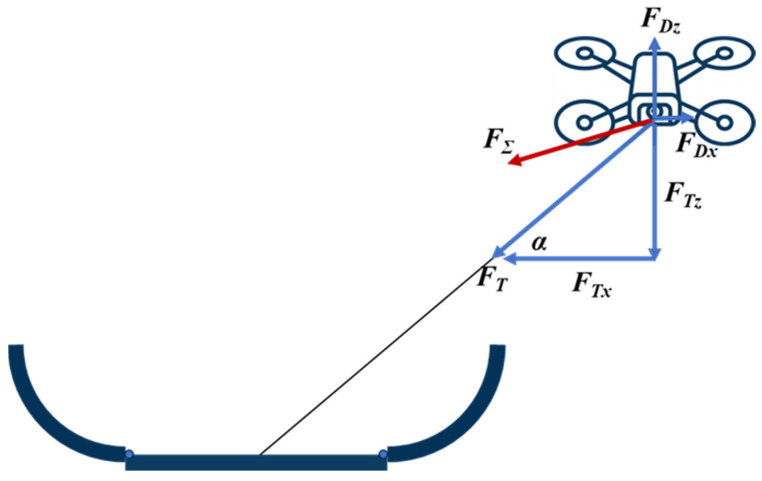
Free-body diagram of the UAV during passive landing. The force generated by the UAV PID controller partially offsets the tether tension, and their resultant determines the direction of motion.

**Figure 9:**
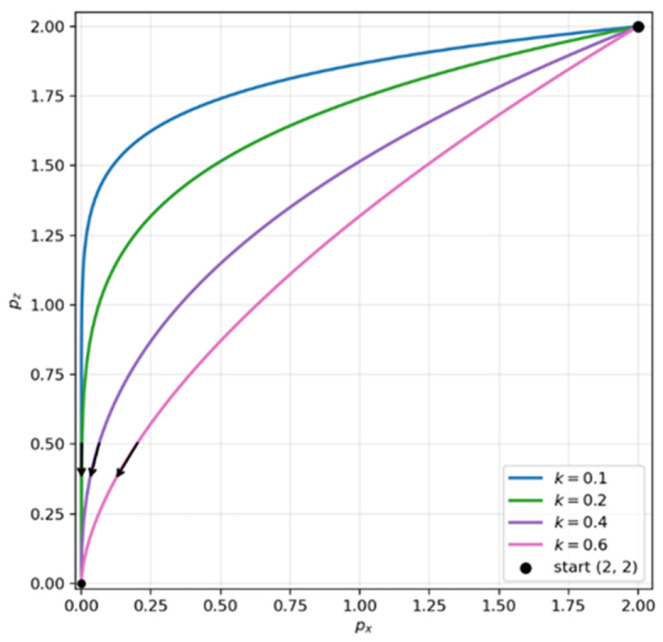
Simulated UAV landing trajectories. As the ratio of horizontal to vertical damping decreases, the UAV increasingly corrects its horizontal position before descending, which is beneficial for passive landing. The arrows indicate the UAV motion direction along each trajectory.

This subsection analyzes the trajectory advantage of passive landing. At the instant the UAV is pulled, the force decomposition shown in Figure 8 applies. Because the tether attachment point in our system is located close to the UAV center of mass, we assume that tether tension does not introduce an additional moment. Let the tether tension be $F_{T}$, with horizontal and vertical components $F_{Tx}$ and $F_{Tz}$, respectively. Let the combined effect of UAV thrust and gravity be $F_{D}$, with components $F_{Dx}$ and $F_{Dz}$. The net force is therefore $F_{\Sigma }$. Although this resultant tends to drive the UAV toward the ground anchor, the motion does not necessarily follow the tether’s direction exactly. When the force generated by the zero-velocity feedback loop, dominated by proportional gain, is approximated as a damping term [50,51], the ideal passive-landing trajectory depends on the ratio of the horizontal and vertical damping coefficients; the derivation is given in Appendix A. Figure 9 plots the ideal passive-landing trajectories for damping-ratio values of 0.1, 0.2, 0.4, and 0.6. As the ratio of horizontal to vertical damping decreases, the UAV tends to correct its horizontal position more aggressively during reeling and align above the anchor before descending. This behavior reduces the likelihood that the vehicle will be blocked by the bay side lid or other structural features during passive landing, thereby facilitating smooth entry into the UAV bay. Accordingly, as described in Section 2.3.2, when designing the PID-based zero-velocity loop, we intentionally increase the gain along the z-axis to increase vertical damping and improve landing success.

#### 3.3.2. Repeated Landing Tests and System-Level Validation

**Figure 10:**
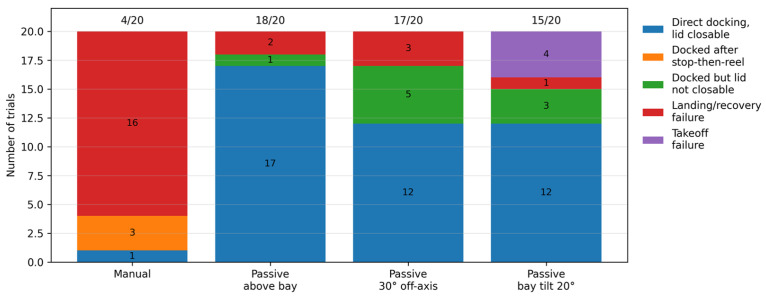
Outcome distribution of the repeated landing tests under four conditions: manual landing, passive landing initiated above the bay, passive landing initiated from an oblique direction of approximately 30°, and passive landing initiated above a bay tilted by 20°. Each bar summarizes 20 trials. The categories are direct docking with lid closure, staged manual success after additional reeling (the UAV descended quickly and required a short additional reeling phase after touchdown), successful docking with a nonideal final pose, landing/recovery failure, and takeoff failure.

This subsection reports both standalone passive-landing tests and integrated flight-validation experiments. To evaluate repeatability under controlled conditions, we performed four indoor test conditions with 20 trials each: manual landing, passive landing initiated above the bay, passive landing initiated from an oblique direction of approximately 30°, and passive landing initiated above a bay tilted by 20°. In the manual baseline, the UAV was first moved to a random position and then landed by the operator; in the passive-landing cases, the UAV likewise started from random positions, but the mode was triggered only after the prescribed terminal relation to the bay was reached. As summarized in Figure 10, manual landing achieved only 4/20 successful recoveries and remained strongly operator-dependent. Passive landing increased the success rate to 18/20 when initiated above the bay and remained effective at 17/20 from the 30° off-axis direction. These results show that tether-assisted recovery without visual fiducials is practically achievable on the proposed platform and can tolerate moderate terminal offset. However, one limitation should be noted: although the self-aligning groove clearly improved pose regularization in the experiments, off-axis recoveries more often ended in configurations from which the UAV could not automatically readjust into the fully aligned pose. When the bay was tilted by 20°, the overall success rate was 15/20. Importantly, most failed trials in this condition occurred during takeoff rather than during landing, because the tilted launch surface introduced lateral velocity before tether release could fully follow the vehicle motion. During passive landing, by contrast, the tether continuously constrained the UAV toward the anchor, while the self-aligning groove guided the landing legs into the customized groove, so moderate touchdown tilt did not readily cause lateral drift.

**Figure 11:**
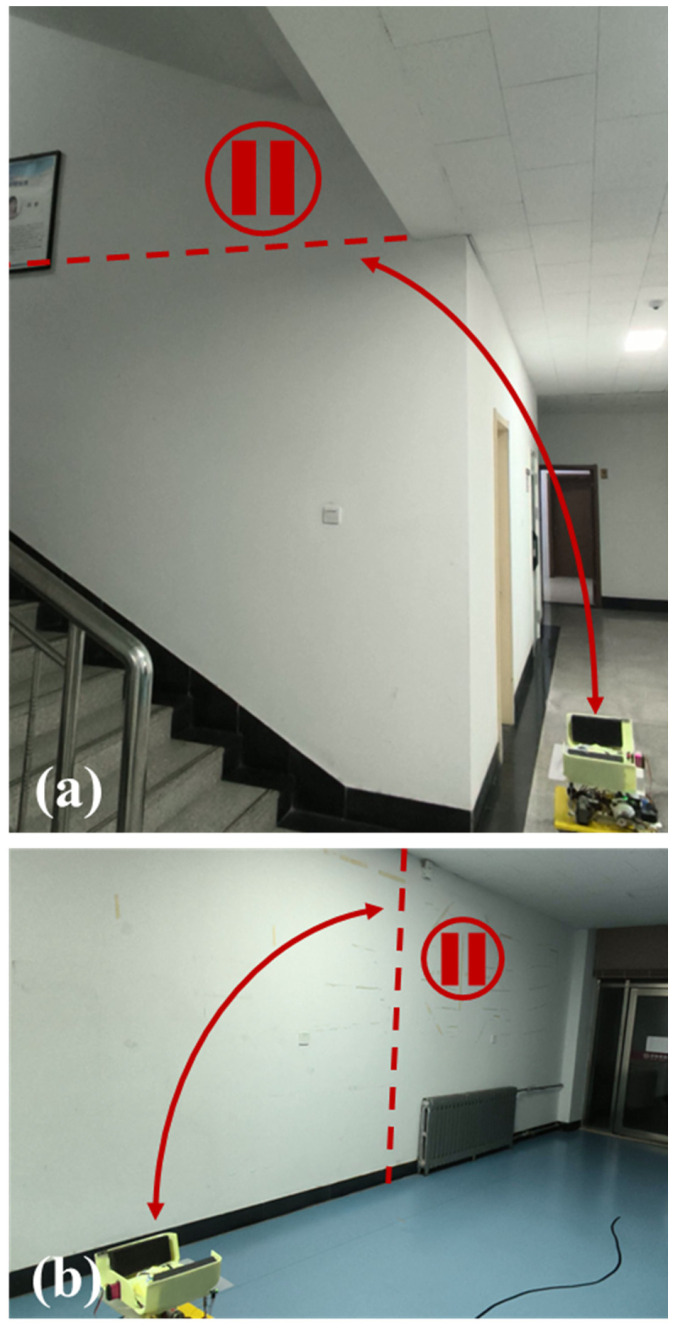
Experimental layouts for system-level flight validation. (**a**) Layout I; (**b**) Layout II. After takeoff, the UAV must cross the dashed line, remain in the designated task region for a short period, and then return for landing. The red curved arrows indicate the UAV flight direction, the dashed lines indicate the predefined boundary, and the pause symbols indicate the task regions where the UAV remains for a short period.

We then conducted system-level validation to verify end-to-end coordination among bay opening, tether payout, the modified flight controller, passive landing, and post-touchdown release. Two representative indoor layouts were used, as shown in Figure 11a,b. In both cases, the UAV took off from the bay, crossed a predefined boundary to leave the immediate vicinity of the station, remained in the designated task region for a short period, and then returned above the bay to initiate passive landing. The two layouts were included to show that the complete operating sequence was not tied to a single departure direction or indoor geometry.

**Figure 12:**
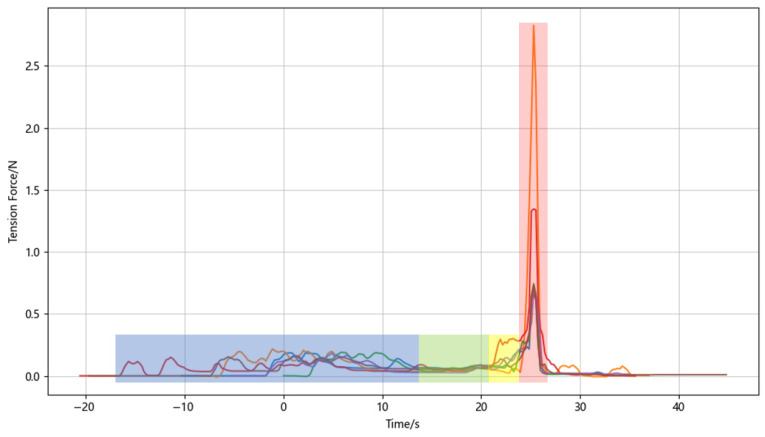
Tether-tension measurements in the system-level flight validation. The four distinct tension patterns clearly correspond to changes in the UAV operating state. The blue, green, yellow, and red shaded regions denote takeoff and outbound flight, task execution and return, passive-landing tether reeling, and completed landing, respectively.

The corresponding tether-tension traces, aligned at touchdown, are shown in Figure 12. During takeoff and outbound flight, the tension fluctuates because the UAV is drawing the tether from the spool. During task execution and return, the signal remains close to zero, indicating that the UAV is no longer pulling out a line. After passive landing is activated, the tension rises again as the vehicle is reeled downward. Finally, a sharp peak appears once the UAV is seated in the bay and the tether becomes taut. This repeatable phase-dependent pattern shows that tether tension in the proposed system is not merely a monitored signal but a practical state indicator that can be used to stop winding and release the clutch at touchdown. In our system, both a maximum tension threshold and a maximum allowable slope for sudden tension increase are defined; if either is exceeded, the system immediately initiates the payout routine, stops the spool motor, and presses the clutch button. These tests should therefore be interpreted as controlled-condition workflow validation rather than outdoor robustness verification.

### 3.4. Exploratory Simulation Toward Ground-Anchor Localization

**Figure 13:**
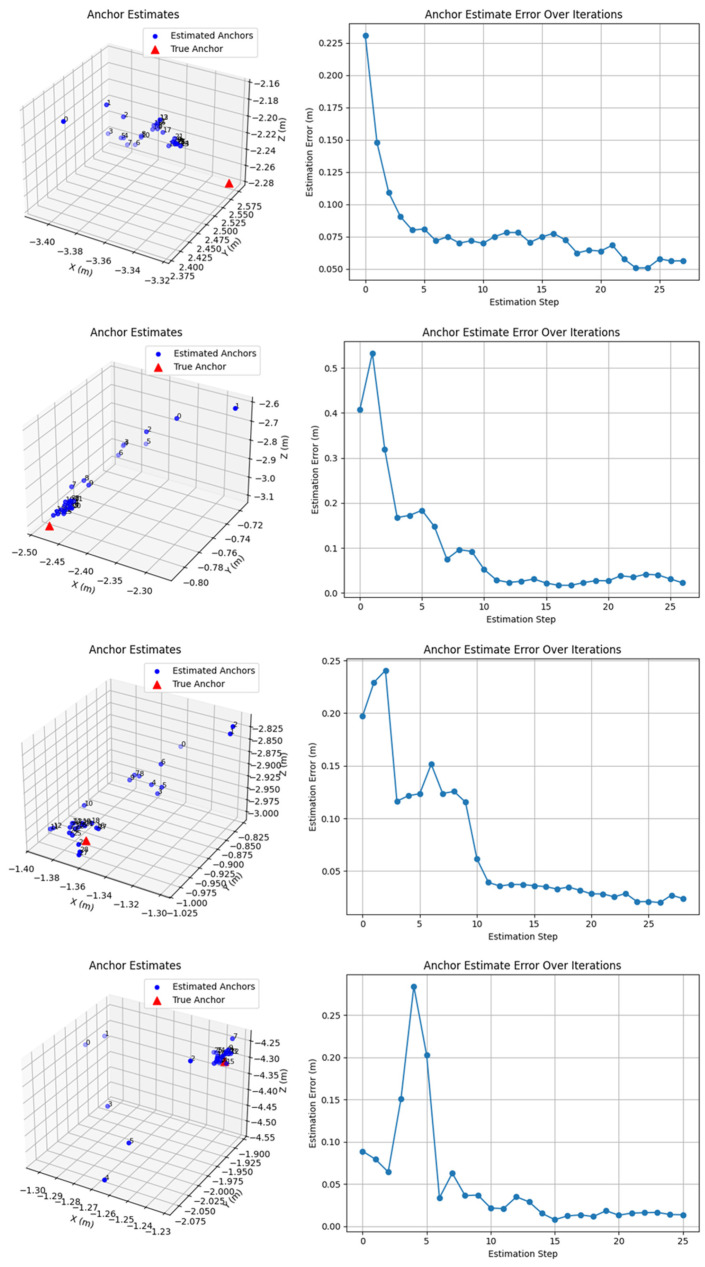
Simplified simulation of the ground-anchor localization algorithm. In each case, the **left** panel shows the true anchor and the estimated anchor positions over successive iterations. The **right** panel shows the distance error between the true and estimated anchor positions versus iteration number, indicating representative convergence behavior under simplified assumptions. The local overlap of estimated points reflects convergence during iteration and does not affect scientific interpretation, because the corresponding scalar distance-error information is shown in the right panel.

This subsection uses simulation to illustrate the broader algorithmic potential of TUAVs. Beyond recovery, the tether may also provide directional information for relative localization. To examine this possibility, we conducted a simplified simulation of a ground-anchor localization concept. In each trial, the UAV was first assumed to hover at a known position, after which a ground anchor was randomly generated within a prescribed spatial range below the UAV. A short disturbance directed toward the anchor was then applied. Using the disturbance direction and the current UAV position, a spatial line was constructed and interpreted as a candidate line passing through the anchor. Repeating this process at multiple UAV positions yielded a set of lines, from which the anchor position was estimated by least squares. More than 50 trials were implemented in Python 3.13.2, and 4 representative results are shown in Figure 13.

These results should be interpreted only as exploratory evidence under simplified assumptions. The current simulation includes only basic gravity, aerodynamic drag, and an initial wind perturbation and does not yet model sensor noise, sustained wind-field disturbances, actuator-dynamic errors, or other real-world effects. Accordingly, the observed convergence behavior and the sub-8 cm terminal errors in some trials should not be regarded as experimentally validated localization accuracy but rather as an indication that the tether-based localization idea is plausible and worth future hardware-based investigation.

In a future physical implementation, the main expected error sources include uncertainty in estimating the disturbance direction from onboard sensing, bias caused by persistent wind or model mismatch, and geometric ill-conditioning when the sampled UAV positions do not provide sufficiently diverse line intersections. These effects are not addressed in the present simulation.

## 4. Discussion

UGV-UAV collaborative systems can provide ground vehicles with an exceptionally valuable aerial perspective, but existing industrial UAV ground stations are generally too bulky and heavy to integrate with small, highly mobile UGVs. To address this problem, we developed a lightweight tethered UAV ground station with both self-locking and self-leveling capabilities. By integrating a 2-DOF self-leveling platform, a custom clutch-enabled reeling mechanism, and a passive-landing control strategy without visual fiducials, the complete system mass is reduced to 4.1 kg. The experimental results show that the platform provides effective launch-and-recovery support under the tested tilted conditions and enables repeatable passive landing without visual fiducials on the proposed prototype.

### 4.1. Lightweight Design and the Value of Aerial Perception

Mainstream commercial tethered ground stations currently weigh approximately 19.5–34 kg, which is already close to or beyond the payload limit of small UGVs. Through a compact layered structure and a highly integrated hardware architecture, our design reduces total system mass to 4.1 kg, or about 20% of that of mainstream commercial systems. Here, the comparison with commercial systems should be interpreted as an illustrative market survey rather than a direct like-for-like benchmark, because commercially available mobile ground stations specifically designed for micro-UAV deployment on small UGVs remain scarce. This degree of lightweighting makes practical integration of a micro-UAV with a small UGV feasible without sacrificing the payload budget needed for other onboard functions. In addition, the viewpoint comparison mainly serves as an application scenario illustration, showing how an elevated aerial perspective complements the limited ground view in obstacle-rich environments. Together with the lightweight design, this clarifies the practical relevance of deploying a micro-UAV from a small UGV.

### 4.2. Self-Leveling Mechanism and Environmental Adaptability

To ensure safe takeoff and landing on uneven outdoor terrain such as slopes, we designed a 2-DOF self-leveling platform based on a central spherical joint and two active branches. Within the allowable error band, the platform can fully compensate for ground tilts of ±12.02 degrees (24.04 degrees total span), and even outside this range, it still provides approximately 12 degrees of additional correction. This design improves launch and recovery reliability and substantially broadens the operating envelope of the collaborative system. More specifically, the self-leveling mechanism reduces the residual platform inclination to a range more compatible with launch and recovery, while the final touchdown is further stabilized by tether guidance and the self-aligning bay geometry.

### 4.3. Passive Landing and Flight-Control Co-Design

One of the central innovations of this study is the passive-landing scheme based on physical tether reeling, which departs from the conventional dependence of precision landing on vision and substantial edge-computing resources. To coordinate the UAV with the ground tether-management system, we replace the original position loop, which anchors the vehicle to a fixed three-dimensional coordinate, with a zero-velocity feedback loop. Consequently, when the UAV is pulled by the tether, it exhibits damping-like rather than restorative behavior, thereby avoiding loss of control caused by accumulated position error. Dynamic analysis and trajectory simulation further show that moderately increasing the vertical damping gain encourages the UAV to align horizontally above the anchor before descending, reducing the risk of collision with the bay edge. Repeated indoor tests and integrated flight validation confirm that, through coordination between the FSM-governed tether controller and the zero-velocity-loop flight controller, the system achieves repeatable passive recovery without visual fiducials on the proposed platform. Specifically, the success rates reached 18/20 when passive landing was initiated above the bay and 17/20 from an approximately 30° off-axis direction, compared with 4/20 in the manual baseline. When the bay was tilted by 20°, the overall success rate was 15/20, and most failures occurred during takeoff rather than during landing. These results indicate that the proposed strategy is practically viable, tolerant to moderate terminal offset, and aided by the self-aligning bay geometry, although final pose consistency is still weaker in some off-axis recoveries.

### 4.4. Potential for Vision-Free Localization

Beyond landing, the tether of a TUAV also contains information relevant to interaction and estimation. In this study, we explored this possibility through a simplified simulation of ground-anchor localization. Under the assumed model, the estimate typically became stable after about 15 iterations, and sub-8 cm terminal errors were observed in some representative trials. However, these results should be interpreted as exploratory simulation results rather than validated localization performance, because sensor noise, sustained wind-field disturbances, actuator-dynamic errors, and other physical uncertainties were not included.

### 4.5. Limitations and Future Work

Although this study demonstrates the feasibility and advantages of the proposed collaborative system, several limitations remain. First, the proposed vision-free ground-anchor localization algorithm has so far been validated only in a Python simulation environment. In a physical system, sensor uncertainty, sustained wind-field disturbances, actuator-dynamic errors, tether elasticity, and geometric ill-conditioning may affect convergence speed and localization accuracy. Likewise, the passive-landing experiments reported here were conducted indoors without wind, and performance under outdoor wind disturbances still needs to be evaluated and improved. Future work will therefore focus on hardware implementation of the localization concept, outdoor experiments under wind disturbances, optimization of bay geometry and terminal-stage control for better final pose consistency, and comparison with alternative landing or tether-management strategies.

## 5. Conclusions

This paper presents a lightweight tethered ground-station solution for integrating micro-UAVs with small UGVs. The platform incorporates a self-locking UAV bay, a 2-DOF self-leveling mechanism, and a clutch-enabled electromechanical tether-management system. By replacing the position loop with a zero-velocity feedback loop and coordinating it with the ground tether controller, the system achieves tether-assisted passive recovery without visual fiducials and substantially improves recovery repeatability on the proposed prototype. Experimental results show that the prototype weighs 4.1 kg, provides static tilt compensation over a 24-degree range, and supports integrated launch-and-recovery operation, including repeated passive-landing tests with terminal offset and bay tilt. A simplified exploratory simulation further suggests that tether-direction information may support future vision-free anchor localization; this part has not yet been experimentally validated. Overall, this work establishes a lightweight design point and a practically viable tether-assisted recovery strategy for micro-UAV launch-and-recovery on small UGVs.

## Figures and Tables

**Table 1:** State-transition table of the spool finite-state machine.

Current State/Event	E1 Actuate Clutch	E2 Rotate Spool	E3 Stop Spool
S0 Spool Stationary and Locked	S1 Spool Free	S2 Spool Winding	N/A ^1^
S1 Spool Free	Ignore	S2 Spool Winding	N/A ^1^
S2 Spool Winding	Error ^2^	Ignore	S0 Spool Stationary and Locked

^1^ Transition not defined, since E3 is only valid in state S2. ^2^ Invalid operation, because the clutch is mechanically locked in state S2.

**Table 2:** Illustrative market survey of representative commercial UAV ground stations. Because we did not identify commercially available mobile ground stations specifically designed for micro-UAV deployment on small UGVs, the listed products mainly target larger UAV classes and are included to indicate the current product gap rather than as a direct like-for-like benchmark.

System Brand and Model	Typical Weight (kg)	Dimensions (L × W × H, mm)	Compatible UAV Class
Elistair Ligh-T 4 [46]	20.0	627 × 475 × 292	Medium UAVs (e.g., DJI M30 ^1^)
Fotokite Sigma [47]	19.5	635 × 483 × 343	Proprietary medium UAVs
Volarious V-Line Pro for DJI M400 ^1^ [48]	19.5	470 × 365 × 190	Medium-to-large UAVs (e.g., DJI M400 ^1^)
DJI Dock 2 [49]	34.0	570 × 583 × 465	Medium UAVs (e.g., DJI Matrice 3D ^1^)
Proposed system	4.1	300 × 300 × 260	Micro-UAVs

^1^ DJI M30, DJI M400, and DJI Matrice 3D are listed as representative compatible UAV examples. They are manufactured by DJI, Shenzhen, China, and were not used in the experiments of this study.

## Data Availability

The minimal dataset supporting the findings of this study is openly available in Zenodo at https://doi.org/10.5281/zenodo.19059925.

## Appendix A. Derivation of the Passive-Landing Trajectory

At the onset of passive landing, let the tether tension acting on the UAV be $F_{T}$, with horizontal and vertical components $F_{Tx}$ and $F_{Tz}$, respectively. Let $F_{D}$ denote the combined effect of rotor thrust and gravity as shaped by the zero-velocity feedback loop, with components $F_{Dx}$ and $F_{Dz}$. The net force acting on the UAV is therefore $F_{\Sigma }$, whose direction determines the initial motion immediately after reeling begins.

For convenience, the component relations below are written in terms of magnitudes. Resolving the net force into the horizontal and vertical directions gives

(A1) $$F_{\Sigma }=(F_{\Sigma x},F_{\Sigma z}{)}^{T}=(F_{Tx}-F_{Dx},F_{Tz}-F_{Dz}{)}^{T}.$$

Under the zero-velocity feedback loop, which is dominated by proportional gain, the control-generated force can be approximated as a viscous damping term [50,51]:

(A2) $$F_{Dx}=c_{x}v_{x},\;\;\;F_{Dz}=c_{z}v_{z}.$$

After a short transient, the UAV enters a quasi-steady regime in which the reeling speed is approximately constant and the net force is close to zero. Hence,

(A3) $$F_{Tx}=F_{Dx},\;\;\;F_{Tz}=F_{Dz}.$$

Let the ground tether anchor be the origin, and let $p_{x}$ and $p_{z}$ denote the horizontal and vertical coordinates of the UAV center of mass, respectively. Because the tether force is collinear with the tether,

(A4) $$\frac{F_{Tx}}{F_{Tz}}=\frac{p_{x}}{p_{z}}.$$

(A5) $$\tan\;\alpha =\frac{p_{z}}{p_{x}}.$$

If the tether is reeled in at a constant speed $v_{T}$, then the UAV velocity component along the tether is also constant:

(A6) $$v_{x}\cos\;\alpha +v_{z}\sin\;\alpha =v_{T}.$$

(A7) $$v_{x}=\frac{dp_{x}}{dt},\;\;\;v_{z}=\frac{dp_{z}}{dt}.$$

Combining Equations (A2)–(A7) yields

(A8) $$\frac{p_{x}}{p_{z}}=\frac{F_{Tx}}{F_{Tz}}=\frac{F_{Dx}}{F_{Dz}}=\frac{c_{x}(dp_{x}/dt)}{c_{z}(dp_{z}/dt)}.$$

Define the damping ratio

(A9) $$k=\frac{c_{x}}{c_{z}}.$$

Equation (A8) can then be rewritten as

(A10) $$\frac{dp_{z}}{dp_{x}}=k\frac{p_{z}}{p_{x}}.$$

(A11) $$p_{z}=Cp_{x}^{k}.$$

Applying the initial condition $(p_{x0},p_{z0}{)}^{T}$ gives

(A12) $$p_{z}=\frac{p_{z0}}{p_{x0}^{k}}p_{x}^{k}.$$

In our design, the horizontal damping coefficient is intentionally chosen to be smaller than the vertical damping coefficient, so 0<k<1. By setting k= 0.1, 0.2, 0.4, and 0.6, and using the initial condition $(p_{x0},p_{z0}{)}^{T}=(2,2{)}^{T}$, we obtain the trajectories shown in Figure 9.
